# Critical Decision-Making Issues in Disaster Relief Supply Management: A Review

**DOI:** 10.1155/2022/1105839

**Published:** 2022-07-04

**Authors:** Ke Ma, Hong Yan, Yusen Ye, Dao Zhou, Dongce Ma

**Affiliations:** ^1^Business School, Sichuan University, Chengdu, Sichuan 610017, China; ^2^School of Management, Zhejiang Shuren University, Hangzhou, Zhejiang 310015, China; ^3^Institute for Disaster Management and Reconstruction, Sichuan University, Chengdu, Sichuan 610017, China; ^4^Michael Page (Shanghai) Recruitment Co., Ltd, Shenzhen Branch, Shenzhen, Guangdong, 518000, China; ^5^Meishan Management Committee of Sichuan Tianfu New Area, Meishan, Sichuan 620000, China

## Abstract

This paper comprehensively reviews the literature related to disaster relief supply management in recent years by taking the perspectives of three critical decision-making issues, i.e., coordination issues, facility location decisions, and inventory decisions. For each decision-making issue discussed, we clarify the barriers of current research papers and identify the major challenges and critical factors that should be considered. In the following, we present the perspectives on the road of coordination between multiple relief actors, characterize the location decisions of relief facilities with a variety of optimization objectives, and emphasize the importance of relief supply varieties and critical factors in the decisions of disaster relief inventories. Future research directions are recommended for further discussions.

## 1. Introduction

After the occurrence of a disaster, a big population of affected victims needs to live on disaster relief supplies, such as food, water, shelters, and medical treatment, which are required to be delivered in a quick, efficient, and effective way. The outbreak of the COVID-19 pandemic has again emphasized fundamental role of relief supply chains [1]. But the unpredictable nature of disasters leaves uncertain messages on relief supply and demand regarding timing, location, and impact which largely complicates disaster relief operations. Prepositioning disaster relief supplies are must to serve as buffer for an efficient and effective response to disasters. Nevertheless, managing disaster relief supplies is never an easy task in the face of the following issues.

### 1.1. Multiple Relief Actors are Involved with Limited Budgets and Resources

Large-scale disaster relief operations will definitely involve a large number of relief actors, from public sectors like government agencies at all levels, military forces, and humanitarian organizations with official backgrounds (e.g., Red Cross), to private sectors, such as diversified nongovernmental organizations (NGOs), religion-based organizations (e.g., Churches), and private firms (e.g., local retailers and service providers). These organizations either operate self-owned warehouses or contract with their suppliers to acquire relief materials. Supplied with multiple sources after disaster strikes, there would arise operational management problems in material convergence, duplication of effort, and/or potential conflicts in operations, which cause not only huge resources waste but also inefficiency in the relief operations [2]. Besides, finding an optimal number of available medical staffs is also a problem of the emergency department [3].

Not all relief actors are capable of prepositioning relief inventories or operating warehouses due to budget constraints [4]. In fact, the majority of NGOs are poorly supported in finance. Their humanitarian assistances live on postdisaster donations, including cash and material donations, which is hysteretic and would be the main source of unsolicited donations. How to time-efficiently and cost-effectively satisfy the demand of affected populations becomes a critical problem in disaster relief inventory management. On the other hand, when procuring from local/domestic or international suppliers to acquire relief supplies, the availability and accessibility of resources would be a big problem, such as supply shortage of local/domestic manufacturers/retailers and long lead-time due to international transportation and personnel organization. The strategy and tactics of optimizing constrained resources to meet as many needs of the victims as possible are the keys to successful disaster relief operations, which is the strength of OR [5].

### 1.2. The Status of the Affected Area is Complicated and Unpredictable

Large-scale natural disasters will destroy the infrastructures in the vicinity, paralyze the transportation network, and impede the delivery of relief supplies. While the warehouses or life-saving roads are devastated, relief materials become inaccessible or unavailable. Relief actors must reorganize their operations and relocate relief supplies to serve the demand of victims. The complicated situations in affected areas trigger accessibility and equity problems in delivering material assistance. Some affected people might be trapped far from the points of distribution (PODs) due to the paralyzed postdisaster transportation. Taking the demographic and socioeconomic characteristics of affected populations into consideration is extremely important but difficult. Moreover, secondary disasters, such as aftershocks and disease outbreaks, dynamically change the status of the affected area. The unpredictable environment in the response process will result in changing demand, supply, and information communication patterns, which upgrades the complexity of managing disaster relief supplies.

### 1.3. Demand is Extremely Uncertain and Ever-Changing

To ensure an effective and efficient response to affected demand, information about the timing, location, type, and quantity of relief supplies is needed as much as possible. However, in the aftermath of disasters, particularly catastrophes, demand information is limited, rough, inaccurate, and hysteretic. It is very difficult to precisely evaluate the impact of the disaster as the disaster environment is ever-changing. There is always no time for good planning to satisfy the demand of affected populations in a cost-effective and time-efficient way. Consequently, disaster relief supply chains may result in excessive waste and emissions, which may harm the local communities and environment in the long run [6].

Since disaster relief is about 80% of the logistics it would follow [7], the critical decision-making issues of disaster relief supplies are worth in-depth understanding and exploring to better support the disaster relief operations. Previous relevant research mostly focuses on the inventory prepositioning (e.g., [8–13]), facility location decisions (e.g., [4, 14–16]), and inventory planning and control (e.g., [17–20]). Balcik et al. [21] review papers that determine the capacity, the time, and the location of disaster relief inventories. They categorize the existing studies according to the planning phrases, i.e., predisaster and postdisaster stages, of a disaster management cycle. Behl and Dutta [22] take a thematic point of view to extensively review the extant literature to reflect the shift in the trend of humanitarian supply chain management. Different from their perspectives, Ye et al. [23] shift the focus from reviewing the literature to identifying the gaps between research and practice to discuss three critical decision themes of disaster relief inventory management. Given that the above three operational problems we have identified would severely complicate the disaster relief supply management, as a supplement, this survey summarizes the state-of-art academic research from the perspective of three decision-making issues, i.e., coordination decisions, facility location decisions, and inventory decisions. We endeavour to cite the majority of relevant publications including journal articles, book chapters, and academic works, mostly published within the past decade, to discuss their point of view focused on these three critical decisions of disaster relief supply management. We also introduce the humanitarian logistics practice highlighted by the Logistics Operational Guide as only practice-based research with both generality and validity considerations can contribute to the humanitarian operations [24]. We use databases such as Web of Science, ProQuest, JSTOR, ScienceDirect, Springer, and Emerald along with Google scholar to search keywords such as “disaster relief supply,” “relief material,” “emergency supply,” “emergency material,” “disaster relief supply chain,” and “disaster inventory prepositioning.” We also cross-reference relevant important studies from cited papers without using the keywords we searched. In the rest of the paper, we review the most relevant papers with respect to the three critical decision-making issues in Section 3, Section 4, and Section 5, and summarize the literature with recommendations for future research directions in the final section.

## 2. Coordination Decisions

Disaster relief supply chains could involve a surprising number of different types of relief actors who undertake material supplying tasks in response to a disaster. These humanitarian organizations include government agencies (e.g., Federal Emergency Management Agency, FEMA) and military forces, international emergency relief organizations (e.g., The [25] local/regional/national social organizations (e.g, the One Foundation of China), religious organizations (e.g., local churches), and private sectors (e.g. local or national-wide retailers and manufacturers). Besiou and Van Wassenhove [26] indicate that the changing role and number of stakeholders, particularly regarding the partner relationships and sector collaborations, have been the most frequently discussed topic in Logistics Cluster Practitioner Conference. Because international and local relief organizations operate on different scales, their roles need to be clearly differentiated. Generally, international relief actors stockpile relief supplies in preparation for relative slow-onset disasters and crises and provide long-term humanitarian aid around the world, whereas local relief actors play the main force to deliver the first wave of emergency supplies as they are much closer and more familiar with the terrain, infrastructure, and demographics of the affected areas. Designing coordination mechanisms between multiple relief actors is always the key to effective and efficient disaster relief supply chain management. However, the majority of studies assume a single decision maker in managing relief supplies [21], whereas the coordination issues lack sufficient understanding from an operational perspective.

### 2.1. Coordination Barriers

There are hundreds of relief organizations participating in the rescue and relief operations of a disaster, particularly of a catastrophe. For instance, over 700 NGOs from more than 40 countries provided emergency assistance to the victims of the Asia Tsunami in 2004 [27]. The difficulties of managing the relief supplies in response to disasters are massively upgraded with uncertain demand, limited logistics capacities, poor information feedback, and multiple decision makers [28]. Therefore, coordination between multiple relief actors meets barriers because of the following reasons:

#### 2.1.1. Limited Local Resources

Apart from inventory prepositioning, relief supplies are acquired from three main sources: global procurement, local procurement, and in-kind donations. Although local procurement has a shorter lead time and lower logistics cost [4], the local supplying capability is at a high risk of being destroyed or damaged if struck by major disasters [29]. Besides, local suppliers are very likely to suffer shortages of satisfying the surging demand [30], which might induce competition between relief actors on the scarce relief resources that consequently raises the price of local supplies [27]. Moreover, supporting resources are also insufficient, such as the increasing demand for vehicles and staff and the extra financial burden on relief organizations or directly on the people in distress [27].

#### 2.1.2. Unsolicited Supplies/Donations

Many postdisaster in-kind donations are spontaneous without knowing the actual demand. These unsolicited supplies/donations from well-wishers always cause congestion in the logistics systems [31]. In the 2004 Indonesia tsunami, 2005 Hurricane Katrina, 2010 Tohoku earthquake, and even in man-made disasters like the 9/11 terrorist attack, it has been highly noticed that unsolicited supplies were donated at the wrong time, in excessive volume, and in unmatchable types to affected areas, especially after sudden-onset disasters [27, 30, 32]. Time and resources are occupied to identify, prioritize, transport, and store these relief supplies, which severely disrupt the priority of material supplying, occupy limited warehouse space, clog the transportation networks, and undermine the relief operations [31]. Therefore, coordination mechanisms are desired to quickly share and publish information about the relief supplies needed and to organize the deployment and transportation within the whole system to avoid congestion and waste.

#### 2.1.3. Poor Communication and Information Sharing

In the face of highly uncertain demand information about the quantity, type, and location of relief supplies, decision makers are difficult to predict the aggregated demand and available resources to supply, resulting in a significant mismatch of demand and supply. For example, 211 million pounds of ice were ordered by FEMA in one week after the landing of Hurricane Katrina and 60% was unnecessary afterward [30]. Relief organizations also have coordination problems in observing each other's operations under asymmetric information. As the postdisaster environment is ever-changing, an affected region is possible to have been repeatedly served. This kind of effort duplication worsens the scarcity of relief resources [2].

Another big challenge the humanitarian decision maker has to continuously deal with is the persistence of coordination-information bubbles. Comes et al. [33] conduct two case studies on Typhoon Haiyan and the Syria Crisis and emphasize the fragmentation and misalignment of coordination structures and decisions in the emergency, which are created by volatile information and sensemaking response. It is imperative to design methods and approaches to help the decision makers identify the role of information in emergent coordination and make adaptive decisions.

#### 2.1.4. Involvement of Government and Military Operations

A larger proportion of the global affected population are residents of developing countries. In developing countries, governments at all levels are the most reliable forces in disaster relief operations. They also take the leading role in coordinating different social organizations and private firms to provide relief supplies effectively and efficiently. Similar to inter-organizational coordination, the necessity of bridging the intergovernmental distance also became recognized explicitly [34]. Once the government fails, people would suffer. Meanwhile, the military is a special and critical relief force that is equipped with more advanced logistics capability and expertise than most relief organizations in deploying a large number of relief supplies. However, many relief organizations are reluctant to cooperate with the military force with the concern of triggering conflicts due to different missions, mandates, working disciplines, and/or operating procedures [27, 35].

In summary, cooperation procedures and coordination mechanisms should be established to clarify the role of each relief actor, to share information (e.g., logistics capacities, real-time emergency supplies, demand estimation, and operation feedback), to identify resource availability and accessibility, to avoid resource duplications and waste, to manage and deploy relief supplies in a coordinated manner, to reduce inventory-related costs and most importantly, to better serve the beneficiaries and mitigate human sufferings. Therefore, we review the literature from the perspective of macro- and micro-coordination to understand how previous research investigates the coordination issues and the corresponding solutions.

### 2.2. Coordination Perspective

Academics and practitioners hold a consensus view that effective coordination between multiple relief actors throughout the disaster relief phases creates the basis for improving logistics performance [23, 36–38]. We discuss the respective macro- and micro-coordination of the disaster relief supply chain in this section.

#### 2.2.1. Macro-Coordination

Macro-coordination refers to establishing a coordination platform on which critical information is centrally gathered and disseminated where the operational standard and guidelines for involved relief actors are set up. Coordination is achieved on three levels: (1) information sharing (e.g., sharing supply and demand information); (2) operational cooperation (e.g., cooperation in transportation or warehousing); (3) organization alliance (e.g., Logistics Clusters). The coordination platform for each level is needed to clarify the partnerships among different relief organizations, integrate collected information on demand and supply, and streamline the utilization of limited resources to avoid duplication of efforts and resource redundancy. In catastrophes, a coordination platform should be designed in a dynamic manner as the complexity of the disaster environment is upgrading. At the national level, the government at all levels usually takes the leading role in the coordination platform.

The coordination platform, for example, the On-site Operation Coordination Centre (OSOCC) established by the UN Disaster Assessment and Coordination Team (UNDAC) and the United Nations Joint Logistics Centre (UNJLC), serves as a focal point for information exchange that facilitates the coordination meetings, demand assessment, and telecommunication, and reports to new arrived relief organizations and coordinates to local authorities [39]. Similarly, the UNJLC also tracks the movement of relief supplies. Such platforms lay a foundation for disaster relief organizations to cooperate, track the movement of goods, and better solve coordination problems of relief supplies.

In addition to the establishment of coordination platforms, widely accepted standards and guidelines for streamlined relief operations are imperative as well. Disaster relief operations will inevitably involve a number of new and inexperienced relief actors in providing emergency assistance where the quality of relief operations might be impaired [36, 40]. Those who (both organizations and individuals) are not capable of following the qualified standard must be rejected. On the other hand, operational guidelines should be provided to encourage the further training of relief organizations and the engagement of private firms. Private firms either contracted with relief organizations in humanitarian logistics or donating relief supplies spontaneously are essential forces in disaster relief operations [41]. They are required to comply with the material supplying principles, collaborate with professional relief organizations, and get familiar with the operational procedures in a disaster relief environment.

The cluster approach for coordination combines “platform” and “standard” in the relief community. Clusters are made up of humanitarian organizations, including UN agencies, NGOs, the Red Cross and Red Crescent Movement, and other social organizations or even government representatives. They collaborate in addressing the needs of a specific sector (e.g., logistics, camp coordination, health, and protection). Clusters provide a framework for actors to jointly respond to the commonly identified needs, design strategic plans with shared objectives, and effectively coordinate both amongst themselves and with the national authorities [42]. Each sector should develop a matchable labor division and set up corresponding operational standards and guidelines [27]. Specifically, the Logistics Cluster is a partner collaboration community aiming at breaking logistics constraints and improving logistics response in the humanitarian environment, which contains four pillars, i.e., partnership base, standards and policy, strengthening response capacity, and operational support [42]. However, the balance of the horizontal coordination inside clusters and the vertical coordination between clusters still waits for further exploration [43].

#### 2.2.2. Micro-Coordination

Since it is impossible for any single relief actor to respond to the disaster, micro-coordination activities are needed between and within relief organizations and private sectors to achieve joint goals.

*(1) Coordination between relief organizations*. Horizontal cooperation is universally observed in practice between multiple relief organizations [23, 27, 44], which contains joint decision-making and collaborative program (e.g., Central Emergency Fund and Consolidated Appeals), cooperation in procurement, transportation, and warehousing, etc. Adida et al. [45] find that regional hospitals with limited budgets collaborate with each other under mutual aid agreements to make decisions on medical inventory stockpiling (e.g., personal protective equipment, such as masks, gloves, and gowns) in case of medical supply shortage. By establishing a supply resource sharing network, Davis et al. [2] conclude that warehousing coordination enables relief organizations to access external supplies in neighbouring warehouses for demand fulfilment.

International relief organizations usually cooperate in resource sharing and joint decision making. For example, as an umbrella organization, the United Nations Humanitarian Response Depot (UNHRD) network provides inventory prepositioning, warehousing, and monitoring services using their warehouses located in Italy, Ghana, United Arab Emirates, Malaysia, and Panama, free of charge, for a wide range of authorized relief organizations [27]. Toyasaki et al. [44] focus on the horizontal cooperation issues of UNHRD members, such as the motive of becoming a member, the optimal coordination mechanism, and the stock rationing decisions of members, in order to propose the optimal inventory management policy for UNHRD. As for resource sharing, Altay [29] introduces a database management tool, i.e., National Incident Management System-Incident Resource Inventory System (NIMS-IRIS), used by signatory states in emergency resources (e.g., aircraft, food, and water) requests. Their analytical model is used on the NIMS-IRIS system to optimally and quickly allocate limited resources and minimize the total cost. Ergun et al. [46] document the use of an IT tool after the 2010 Haiti earthquake to improve last-mile supply distribution and data management in the camp management. They also introduce a cooperative game theory model, which is motivated by practical examples, and develop the conditions under which multiagency coordination is feasible and desirable. Li and Wang [47] use the scenario construction technology to design the emergency management system of urban flood, which is artificially intelligent in mobilizing and coordinating functional departments to facilitate the establishment of emergency management system and the standardization of operation procedures.

*(2) Coordination with private logistics service providers*. An increasing number of private firms, particularly those logistics service providers, have participated in disaster relief operations. Examples of such long-term partnerships can be found between WFP and TNT, the American Red Cross and FedEx, and Mercy Crops and DHL [27]. The long-term partnership is possible to evolve into alliances, such as the collaboration among Quality Medical Donations, the Disaster Resource Network, and the Business Roundtable [48], which is beneficial to serving ultimate beneficiaries.

The important role of private firms has been highlighted by both literature and practice, whereas the potential threats and challenges of collaborative partnerships cannot be omitted. While relief organizations outsource transportation or food supplying to private firms, there poses a potential risk of breach of contract if private firms lose profits or have security concerns. The partner relief organization might have to bear huge humanitarian losses that cannot be compensated. Egan [41] proposes three solutions with or without marketing approaches to solving the problems of contracting failure and over-reliance on private sectors.

Table 1 summarizes the coordination mechanisms in disaster relief supply management from respective marco- and micro-coordination perspectives.

## 3. Facility Location Decisions

In the disaster environment, where to preposition the relief supplies before and after the occurrence of disasters significantly affects the performance of disaster relief operations. The location decision metrics between the (potential) disaster sites and the selection of facilities concern: (1) which warehouses or distribution points should be utilized or established; (2) which disaster sites should be served by the selected facilities. This section discusses how the literature selects and establishes facilities to store relief supplies according to the hierarchy of facilities so as to optimally determine the number, the location, and the capacity of facilities.

### 3.1. Facility Hierarchy

Perspectives from both time and space dimensions are most commonly hired to define the facility types in the facility hierarchy to describe the material flows. From the temporal perspective, warehouses or distribution centers established for long-term stockpiles of relief supplies are defined as permanent facilities, such as FEMA logistics centers and the UNHRD. Temporary distribution points are located much closer to the disaster affected regions, such as State Staging Areas (SSA) of FEMA, local rescue centers, Points of Distribution (PODs) (e.g., local schools, big parking lots, sports centers, and churches), also including supply ships and mobile vehicles [16]. On the other hand, the spatial perspective categorizes facilities as regional/national and local facilities. Regional/national facilities are usually set up by the government or international relief organizations to cover widespread areas. FEMA logistics centers and UNHRD both fall into this category. Local facilities, by contrast, have much smaller capacities, such as local distribution/rescue centers, regional rescue centers, and Break of Bulk points (BOBs), as they serve relatively smaller regions. Take FEMA's relief network for instance. Seven components are included, which are FEMA logistics centers, Commercial Storage Sites (CSSs), other Federal Agencies Sites (VEN), Mobilization (MOB) Centres, Federal Operational Staging Areas (FOSAs), SSAs, and PODs [49]. Only FEMA logistics centers and CSSs are permanent and national facilities, whereas the rest are temporary and local ones that are set up or deployed according to the demand requirements after disaster strikes. The Logistics Operational Guide has identified the key points of regional facility decision makings, which are readily available access to a high volume of intermodal international transport, relative location to the area of response, the nature of planned interventions, political climate of the country, economic feasibility, access the correct amenities, and access to sufficient technical support.

Many related papers have explored more than one layer of the facility hierarchy to make their models realistic. For instance, Balcik et al. [50] propose a three-layer distribution network including the primary hub (seaports or airports), the secondary hub (permanent central warehouses), and the tertiary hub (local distribution centers). Görmez et al. [51] propose a hierarchical facility location problem and initially locate the temporary facilities. Döyen et al. [52] build a two-echelon stochastic model to determine the locations of uncapacitated regional rescue centers and capacitated local rescue points. Noyan et al. [53] include both local distribution centers and PODs in the relief network to preposition and distribute relief items. To facilitate remote victims to get access to relief supplies, Horner and Downs [54] introduce BOBs which are designed with inferior infrastructure requirements compared to those of the PODs but are chosen closer to remote disaster sites that enable PODs to be located in the vicinity of big population centers. While using scenarios in the model, Mete and Zabinsky [55] study where to preposition additional medical supplies for potential earthquakes based on the existing network of hospital warehouses in Seattle. Bozkurt and Duran [56] provide suggestions for CARE international about how to expand the world-wide prepositioning network of relief supplies. Klibi et al. [57] propose a three-echelon relief network consisting of distribution centers, PODs, and vendors to simulate real-life emergency relief operations and system performance.

### 3.2. Facility Location

The extremely uncertain and complex disaster environment, which is greatly different from the commercial context, would frustrate the relief supply decision makers. While those facility location models with business context cannot well apply in disaster context [58], disaster relief inventory prepositioning needs to make decisions on the number (for each layer if considered), the location, and the capacity of facilities by properly evaluating the following critical factors in the optimization models.

#### 3.2.1. Beneficiary Service Level

To alleviate the sufferings of vulnerable people, relief supplies are required to be delivered to the ultimate beneficiaries efficiently, effectively, and impartially. The optimization issues regarding response time, demand coverage, and equity are on the top of the location-decision list. A majority of studies consider the minimization of response time as their major optimization objective. Duran et al. [10], Bozkurt and Duran [56] and Rezaei-Malek et al. [59] seek to minimize the average of the weighted response time, where the weights are chosen according to the proportions of the realized demand flow, to optimally determine the number and the locations of prepositioning warehouses. Renkli and Duran [60] minimize the response time by minimizing the total weighted delivery distance, and Tofighi et al. [61] simultaneously minimize the total distribution time and the maximum weighted distribution time of critical items to determine the locations and the inventory levels of both central and local warehouses. To measure the performance, Balcik and Beamon [4] set up upper and lower bounds of response time in their maximal covering model to choose the qualified number, location, and stocking levels of capacitated distribution centers.

Maximizing the coverage of relief demand is a big challenge in choosing facility locations because as the disaster environment changes overtime, the type, amount, and location of the affected population are difficult to estimate. Unfortunately, there is always a proportion of demand that cannot be well satisfied, which is defined by Jia et al. [14]; Mete and Zabinsky [55]; Rawls and Turnquist [62]; Rezaei-Malek et al. [59] and Tofighi et al. [61] as unmet/unfulfilled demand and is measured as penalty cost in the model to minimize. Other related research addresses the demand for covering problems by using different objectives. Tean [63] maximizes the number of expected disaster survivors. Balcik and Beamon [4] and Mohammadi et al. [13] maximize the total expected demand coverage in their models. Afshar and Haghani [49] and Van Hentenryck et al. [64] choose the minimization of unsatisfied demand as one of their model objectives in the location decision.

In terms of the equity problem in delivering relief supplies, Noyan et al. [53] concern about the mobility of affected people which may alter their accessibility to relief supplies, as well as the impartiality issues to achieve high-level equity during emergency response. Equity is also considered by Mohammadi et al. [13] in their model to provide an equal amount of relief items to all demand nodes in case of discrimination.

#### 3.2.2. Humanitarian Logistics Cost

Controlling the logistics cost is also important to relief sectors because logistics cost can contribute a large proportion, up to 80%, of the total operational cost. [65]. Given that most NGOs have limited transportation capacity and financial budget (donations are generally launched after disasters), how to control the cost of locating facilities without degrading the service level in emergency response is a vital problem for relief organizations to solve.

Balcik and Beamon [4] consider the cost of establishing distribution centers as well as acquiring and prepositioning relief supplies under budget limits in the predisaster phase. Manopiniwes et al. [66] determine the location of warehouses by jointly considering time and capacity constraints to minimize the total logistics cost, including the opening cost of warehouses, in the flood relief operation in Thailand. Mohammadi et al. [13] determine the optimal number and location of distribution centers (DCs) used for prepositioning relief supplies against earthquakes with the minimization objective of the total cost of establishing DCs, acquiring, storage, and transportation. Rezaei-Malek et al. [59] determine the locations of warehouses to minimize the total operational cost together with the response time in their model. More broadly, the logistics cost is minimized in other ways. Van Hentenryck et al. [64] examine the cost of prepositioning relief items in selected warehouses, and Horner and Downs [54] minimize the distribution cost of developing a relief network, while Mete and Zabinsky [55] put the total operating cost of medical warehouses into the objective function of their stochastic programming model. Both Tofighi et al. [61] and Rezaei-Malek et al. [59] consider the additional cost of unused relief inventories in the preparation of disaster relief operations. Chu and Chen [67] design a novel effective IFA-GA algorithm to solve a multiobjective optimization function which contains the deprivation cost, the unsatisfied demand cost, and the logistics cost.

#### 3.2.3. Infrastructure and Transportation Network

Infrastructure issues focus on the facility conditions of warehouses and distribution centers, roads, and transportation lanes, which are at the risk of being destroyed by a disaster. Therefore, location decisions must involve the potential disruptions of infrastructure and transportation networks which undermines the disaster relief operations. Ukkusuri and Yushimito [68] preposition relief supplies by considering the reliability of transportation networks in disasters. They choose supply holding locations to deliver relief supplies to demand points with maximal probability. Likewise, Hong et al. [69] define “network reliability” as the probability of a possible flow of relief supplies in the postdisaster phase. They introduce global and local probabilistic constraints to realize high reliability of the relief network. Renkli and Duran [60] examine the survivability of infrastructure to formulate uncapacitated locations of relief facilities. Paul and MacDonald [70] take the possible damage of earthquakes into account when they determine the initial capacity of DCs in the preestablished network.

Another stream of research sets up selection criteria for facilities. Kapucu et al. [71] categorize different types of the staging area. They list 10 general criteria to guide emergency government agencies to select staging areas from candidate sites, including location (e.g., at a safe distance from disaster sites), operation center location, highway/road access, helicopter access, safety and security, demobilization, hardstand, equipment, storage, and utilities. Roh et al. [72] identify five groups of selection criteria in facility location decisions, including location, logistics, national stability, cost, and cooperation. They show that the subattributes “political stability” and “economic stability” from the national stability group are the most significant factors for the location selection of relief warehouses.

All the critical factors and the corresponding measurement of each group for the location decisions of disaster relief facilities are summarized in Table 2.

## 4. Inventory Decisions

Critical inventory decisions for disaster relief supplies are made on the inventory level, order quantity, reorder point, etc. (see [17, 73, 74]. Because both demand and supply are highly unpredictable and ever-changing during disaster relief operations, inventory decisions of relief supplies are also dynamic, posing a great challenge to relief actors.

### 4.1. Relief Supply Variety

We first characterize a variety of different types of disaster relief supplies as the inventory decisions must be made specifically for a group of relief items. Living necessities of affected populations cover a wide range of relief items such as bottled water, instant or can food, clothes, sanitation equipment, medicine, shelters, and tents [58, 75]. Among these, food is a focused type that must be well managed and properly replenished due to its perishability [76, 77]. Salas et al. [78] investigate the inventory problem of perishable food by using a stochastic programming model to minimize all related costs including ordering, shortage, disposal, and penalty cost. Natarajan and Swaminathan [79] consider UNICEF's financial constraints in procuring ready-to-use therapeutic food (RUTF) to maintain their relief inventory level in Africa. Kunz et al. [80] analyze the delivery of RUTF during the postdisaster response phase to figure out the impact of disaster management capability (DMC) on lead time. Besides, medical supplies are also an imperative type of relief supplies for life-saving. Mete and Zabinsky [55] focus on how to preposition medical supplies that need to be distributed to hospitals following a disaster. Tofighi et al. [61] put medical first-aid kits into the critical relief items to be kept in both central warehouses and local distribution centers. Moreover, given the poor sanitation conditions of the disaster sites, the demand for some typical medicine raises up in case of a secondary outbreak of diseases, such as Artemisinin Combination Therapy (ATC) for malaria [81].

Relief inventory items also contain various durable products. Such types of items are identified by UNHRD, which consist of electrical devices, individual kits, office and living accommodation, radio and telecommunication, shelters and housings, and water supply systems. [82]. Taskin and Lodree [18] study manufacturers' inventory control responding to generator orders placed by relief organizations and government agencies, revealing the objective conflicts between the manufacturers and public sectors during the Hurricane season. Lodree [83] categorizes two types of relief supplies: (1) seasonal products like flashlights and generators; (2) consumable products like long shelf-life food and bottled water. Some papers also look into other disaster-related inventory types, such as spare parts inventory of trucks that carry relief commodities to disaster sites [84] and maintenance components (e.g., generators, transformers, and capacitors) used to recover the electronic power system destroyed by the Hurricane [85]. De Leeuw et al. [86] investigate the stockpile problem of the Water Sanitation and Hygiene (WASH) cluster (identified by UN) and list a bunch of materials and equipment that support the disaster relief operations in this cluster, including bladder tanks, pipes, pumps, and water purification items, latrine slabs, and potties.

### 4.2. Critical Inventory Decision Issues

As the disaster environment changes over time, relief inventory decisions are affected or restricted by many highly unpredictable issues.

#### 4.2.1. Disaster-Related Uncertainties

Lodree and Taskin [87] specifically consider the initial responses of all relevant disaster relief actors and characterize two types of uncertainties in the optimization model, i.e., the occurrence probability of a disaster and the demand surge for relief supplies, facilities, and human resources. Garrido et al. [88] choose the flood's intensity level and the occurrence probability of a disaster as random input variables of their spatiotemporal stochastic model to determine the optimal inventory level of relief supplies with the objective of maximizing demand satisfaction. Saputra et al. [89] develop a trade-off model and use it in a spreadsheet-based platform to study how the mean time between two disasters affects the strategies for inventory prepositioning. Roni et al. [90] consider both regular and surge demand in the disaster response stage and formulate a new mixed-integer programming model based on the level crossing theory to develop a hybrid policy for disaster relief inventories.

#### 4.2.2. Forecast Information

Some disasters are relatively predictable, such as hurricanes. Then, forecast information has a powerful impact on choosing proper inventory levels of relief supplies. Lodree and Taskin [91] formulate an optimal stopping problem with a Bayesian update framework for manufacturing and retail firms to confront the surging demand for emergency supplies. They refer to the Hurricane predictions to decide when to postpone the emergency inventories in case that tropical depression or disturbance evolves into a severe Hurricane. A stochastic programming model is introduced by Taskin and Lodree [18] to design a proactive inventory policy (on optimal ordering/replenishing points) for the manufacturers and retailers during the prehurricane season, by using historical data to predict seasonal demand.

#### 4.2.3. Financial Constraints

McCoy and Brandeau [92] investigate the relationship between the stockpile size of relief items and the benefit to disaster victims. They give advice to UNHCR on how to choose the optimal inventory level and allocate financial resources with a limited budget. Natarajan and Swaminathan [79] find that the uncertainties in funding timing and funding level of donors have an influence on the relief organizations' operational costs and fill rates. They formulate a multiperiod stochastic inventory model to decide the optimal replenishment policy and to show that avoiding funding delays is critical in a fully funded system, where the front-loaded funding at 75% level supports the disaster relief operations equally as the back-loaded full funding.

#### 4.2.4. Unforeseen Disruption

Disaster relief life cycle is generally divided into two phases in the existing literature, i.e., the preparedness and the response phases [93]. When responding to a disaster, many unforeseen disruptions, such as the outbreak of epidemic, aftershocks, or overlapping disasters (e.g., Hurricane Tomas arisen by the Haiti earthquake), cause additional surging demand within the affected region [81, 93]. The supply shortage is always blamed on the damage to roads, destroyed warehouses, fire, or theft [93, 94]. All of these unforeseen disruptions must be dynamically considered in the inventory plans to satisfy the overall demand of victims. Thereafter, a majority of research focuses on the relocation or transshipment of relief supplies from other functioning warehouses or supply holding ships in the vicinity to reduce the replenishment lead time [94, 95]. Ozguven and Ozbay [96] develop a pLEPs algorithm to solve their inventory control model (MC-SHIC) by determining the inventory levels of relief items in shelters. They collect information about transportation tracking, relief commodity flow, and inventory level fluctuation by RFID technology. Ozguven and Ozbay [97] also improve their previous model by integrally considering the closed-loop feedback-based inventory control that uses RFID devices.

Regarding inventory mobility, Rottkemper et al. [81] propose a mixed-integer programming model to relocate the inventory with the objective of minimizing unsatisfied demand and operating costs. Sarder and Iqbal [94] focus on the relocation of medical relief items for healthcare organizations and propose a three-layer model to minimize the unmet demand and the transportation cost of the system. Mulyono and Ishida [98] format shelter clusters in disaster sites that use the stable roommate (SR) algorithm to build interconnections between shelters. Stock transshipment is implemented in their model in case of a potential shortage of relief items. Kessentini et al. [99] identify the urgent request from some relief centers and provide transshipment options to minimize lead time. They develop an agent-based model and a simulator to solve this problem. Richter [16] proposes dynamic relocation models to give insights on how to relocate the mobile relief inventories carried on a supply ship (e.g., Floating Doctors, Project Hope) in response to the changing demand.

## 5. Conclusions and Future Directions

This paper comprehensively reviews the literature related to disaster relief supply management in recent years, by taking the perspectives of three critical decision-making issues, i.e., coordination issues, facility location decisions, and inventory decisions. For each decision-making issue we discussed, we summarize the current research papers and identify the major challenges and critical factors to be considered. We first clarify the barriers to the road of coordination between multiple relief actors and take both macro- and micro-perspectives on the coordination issues. Then, we emphasize the location decisions of different types of relief facilities with a variety of optimization objectives. After that, we characterize the relief supply variety and discuss the critical factors, such as disaster-related uncertainties, forecast information, financial budget, and unforeseen disruptions, which would have a remarkable influence on the disaster relief inventory control.

Although the importance of coordinating multiple relief actors is realized and highlighted while many cases and coordination mechanisms are mentioned, relief organizations collaborate and cooperate with each other in benefit sharing, and risk-taking is not well addressed in the literature. Besides, criteria to evaluate the performance of relief operations coordination are not well established. In future research, the evaluation criteria must be set up to clearly understand the purposes and consequences of coordination in disaster relief inventory management, as well as to provide guidance and principles for the relief actors to follow to enhance the overall performance.

Another question to be explored is how the suppliers of relief supplies develop their inventory policies in disaster relief operations. Most research focuses on the inventory prepositioning of relief actors in response to disasters. In fact, the suppliers of these relief actors, whose inventory capacities are assumed to be sufficient and assessable as backup resources, must build close partnerships with relief actors to guarantee the material supply. However, they are also at the risk of being destroyed or breaching a contract. Some papers have considered the inventory policies designed for suppliers (see [18, 91]), but the interaction between the suppliers and the relief actors requires in-depth discussions to achieve better-integrated performance and control the potential risks.

## Figures and Tables

**Table 1:** Coordination mechanisms in disaster relief supply management.

Perspective	Mechanism	Example
Macro-coordination	Clusters/platform	Logistics Cluster/OSOCC/UNJLC
	Network	UNHRD
	Standards and guidelines	Operational guidelines/material supplying principles

Micro-coordination	Joint decision making/Collaborative program	Central emergency fund and consolidated appeals
	Resource sharing	Regional hospitals/UNHRD members
	Information system integration	NIMS-IRIS
	Contracting	WFP and TNT/the American Red Cross, andFedEx/Mercy Crops and DHL

**Table 2:** Critical factors in the location decision of disaster relief facilities.

Factor	Measurement	Publication
*Response time*	Average of the weighted response time	e.g., Duran et al. [10]; Bozkurt and Duran [56]; Rezaei-Malek et al. [59]; Tofighi et al. [61]
	Total transportation/distribution/delivery time	
	Total weighted distance	

*Cost*	Budgetary constraints	e.g., Horner and Downs [54]; Mete and Zabinsky [55]; Van Hentenryck et al. [64]; Manopiniwes et al. [66]; Mohammadi et al. [13]; Rezaei-Malek et al. [59]; Tofighi et al. [61]
	Operational cost (storage and distribution)	
	Cost of establishing/opening warehouses	
	Cost of unused inventory	
	Total cost of operating warehouse	

*Demand*	Unmet/unsatisfied demand	e.g., Jia et al. [14]; Mete and Zabinsky [55]; Rawls and Turnquist [62]; Tean [63]; Balcik and Beamon [4]; Mohammadi et al. [13]; Afshar and Haghani [49]; Van Hentenryck et al. [64]
	Total expected demand covered	
	Shortage cost of unmet demand	

*Infrastructure*	Reliability of transportation	e.g., Ukkusuri and Yushimito [68]; Hong et al. [69]; Renkli and Duran [60]; Paul and MacDonald [70]; Kapucu [71]; Roh et al. [72]; Chu and Chen [67]
	Potential damage to DCs	
	Survivability of infrastructure	
	Staging area selection criteria	

*Other factors*	Equity	e.g., Noyan et al. [53]; Mohammadi et al. [13]
	Accessibility	
	Changing trend of natural disasters	
